# Actions and processes that patients, family members, and physicians associate with patient- and family-centered care

**DOI:** 10.1186/s12875-019-0918-7

**Published:** 2019-02-25

**Authors:** Clarissa Hsu, Marlaine Figueroa Gray, Lauren Murray, Marie Abraham, Wendy Nickel, Jennifer M. Sweeney, Dominick L. Frosch, Tracy M. Mroz, Kelly Ehrlich, Bev Johnson, Robert J. Reid

**Affiliations:** 1 Kaiser Permanente Washington Health Research Institute, 1730 Minor Ave., Ste. 1600, Seattle, WA 98101 USA; 2 National Partnership for Women & Families, 1875 Connecticut Ave., NW, Ste. 650, Washington, DC, 20009 USA; 3 Institute for Patient- and Family-Centered Care, 6917 Arlington Road, Ste. 309, Bethesda, MD 20814 USA; 4 Center for Patient Partnership in Healthcare, American College of Physicians, Washington, DC, USA; 5 X4Health, Washington, DC, USA; 6 Palo Alto Medical Foundation Research Institute, 795 El Camino Real, Palo Alto, CA 94301 USA; 7 Department of Rehabilitation Medicine, University of Washington, 325 Ninth Avenue, Box 359612, Seattle, WA 98104 USA; 8 TrilliumHealth Partners, 100 Queensway West, 6th, Mississauga, ON L5B 1B8 Canada

**Keywords:** Qualitative research, Health care improvement, Patient-centered care, Ambulatory care, Health care performance measurement, Clinical training

## Abstract

**Background:**

Patient- and family-centered care (PFCC) is increasingly linked to improved communication, care quality, and patient decision making. However, in order to consistently implement and study PFCC, health care systems and researchers need a solid evidentiary base. Most current definitions and models of PFCC are broad and conceptual, and difficult to translate into measurable behaviors and actions. This paper provides a brief overview of all actions that focus group respondents associated with PFCC in ambulatory (outpatient) care settings and then explores actions associated with the concept of “dignity and respect” in greater detail.

**Methods:**

We conducted nine focus groups with patients, family members, and physicians in three metropolitan regions across the United States. Group discussions were transcribed and analyzed using a thematic analysis approach.

**Results:**

We identified 14 domains and 47 specific actions that patients, family members, and physicians associate with PFCC. In addition to providing a detailed matrix of these domains and actions, this paper details the actions associated with the “dignity and respect” concept. Key domains identified under “dignity and respect” include: 1) building relationships, 2) providing individualized care, and 3) respecting patients’ time. Within these domains we identified specific actions that break down these abstract ideas into explicit and measurable units such as taking time, listening, including family, and minimizing wait times. We identified 9, 6, and 3 specific actions associated, respectively, with building relationships, providing individualized care, and respecting patients’ time.

**Conclusions:**

Our work fills a critical gap in our ability to understand and measure PFCC in ambulatory care settings by breaking down abstract concepts about PFCC into specific measurable actions. Our findings can be used to support research on how PFCC affects clinical outcomes and develop innovative tools and policies to support PFCC.

**Electronic supplementary material:**

The online version of this article (10.1186/s12875-019-0918-7) contains supplementary material, which is available to authorized users.

## Background

Making health care more patient- and family-centered is an increasingly important goal for a range of stakeholders including private and government health care organizations, payers, research funders, patients and families, patient-advocacy groups, and clinician stakeholders [1–6]. A growing body of evidence links improvements in patient- and family-centered care (PFCC) to improved communication and quality, better patient decision making, decreases in unnecessary procedures, and improvements in care efficiency [7–11]. A challenge to improving and providing an evidentiary base for PFCC practices is the lack of consensus on how to define and measure PFCC [12]. There are numerous PFCC definitions and models, but most are broad and conceptual, and difficult to translate into measurable behaviors and actions. Systematic reviews have recently mapped the themes and concepts across PFCC definitions [2, 13–16]. Common concepts include dignity and respect [2, 16, 17], individualized care [16, 18, 19], patient and family engagement, information sharing [2, 17, 18], emotional support [6], and access to care [15, 20, 21]. However, we still lack consensus regarding how best to translate these concepts into practical measurement tools to support PFCC research, improvement, and evaluation [12, 22, 23].

This paper is the result of a collaboration between the American College of Physicians, the Institute for Patient- and Family-Centered Care (IPFCC), the National Partnership for Women & Families, and Kaiser Permanente Washington Health Research Institute. The goal of our collaboration was to develop a behavior-based survey instrument to measure PFCC in primary care and other ambulatory care settings (such as outpatient specialty care). The survey was collaboratively developed by a team of patients, clinicians, and researchers. We began with a series of focus groups to elicit behaviors and actions that patients, family members (defined broadly to include friends and caregivers), and physicians associate with PFCC. We provide a brief overview of all actions respondents associated with PFCC in ambulatory care settings. Because of its particular saliency to patients and families, the actions and behaviors associated with the PFCC concept of “dignity and respect” are described in detail in this paper. These findings fill a critical gap in PFCC research by making abstract concepts about PFCC more concrete, observable, and measurable.

## Methods

We conducted nine focus groups with 92 participants to understand how U.S. patients, family members, and physicians describe ideal PFCC, emphasizing details about specific actions.

- Sampling/recruitment

To increase diversity in respondents’ experiences and attitudes, we conducted focus groups in three metropolitan regions—Greater Seattle, Greater Dallas, and Greater Philadelphia. In each region, we conducted separate focus groups with patients, family members, and physicians who spent at least half of their time working in outpatient settings (includes primary care, urgent care and specialty care).

To recruit patient and family caregivers, we contracted with local market-research firms that used a screening questionnaire we developed. We recruited physicians through the American College of Physicians membership. The Additional file 1 have recruitment details and surveys.

b. Data collection

Two research team members with experience facilitating focus groups and conducting qualitative research (CH and MFG) attended all focus groups. Participants received a meal and a cash incentive of $100.00 for patients and caregivers and $150.00 for physicians. Facilitators structured group discussions around a series of semi-structured questions that were developed to elicit stories about actions that participants felt were patient and family centered and to facilitate brainstorming around the specific actions they would associate with key concepts found in PFCC definitions (Additional file 1). Input about the questions was received from the entire research team as well as an advisory committee that included patients, clinicians, and researcher stakeholders. The interview guides were customized to each type of respondent—patients, family caregivers and physicians. Discussions lasted approximately 2 h for patients and family caregivers and 1.5 h for physicians.

c. Data analysis

The analysis team (MFG and CH) reviewed transcripts and the primary coder (MFG) drafted an initial code list using an inductive analysis approach that involves reading through the data and identifying themes that emerge organically from the data [24]. Because the interview guides emphasized eliciting actions respondents associated with PFCC, the majority of the emergent codes were specific actions that we organized under a priori domains. Both analyzers then coded a patient transcript using the draft code list, compared the results, discussed discrepancies and added and/or refined the codes to ensure that the codes were comprehensive and that the definitions were clear. After comparison, the process was repeated with caregiver and physician transcripts. After three rounds of iterative code development, the primary coder coded all transcripts using final codes and definitions. Atlas.ti was used to assist with coding and data management.

Once coding was completed, the full research team discussed key themes of interest and the organization of the codes. To develop an overall synthesis of the data, we extracted all relevant codes. The lead author (CH) reviewed the data with assistance from another team member (LM). We documented the synthesis with supporting codes in a coding memo. We organized findings in three levels: 1) PFCC concepts (based on the definition IPFCC, Table 1) [17]; 2) domains; and 3) actions. The coding memo and table were shared with all authors for input and feedback regarding the organization and language of data-derived domains and themes.

**Table 1:** IPFCC’s* Definition of Patient- and Family-Centered Care: History & Four Concepts [4, 17]

History/Background	The core concepts of patient- and family-centered care were initially developed in the 1980s by patients, families, clinicians, researchers, and health care leaders. They were grounded in the conceptual frameworks of the consumer and family support movements that gained momentum in health care, social services, and education in the 1960s.
Core Concept	Explanation
*Dignity and Respect*	Health care practitioners listen to and honor patient and family perspectives and choices. Patient and family knowledge, values, beliefs and cultural backgrounds are incorporated into the planning and delivery of care.
*Information Sharing*	Health care practitioners communicate and share complete and unbiased information with patients and families in ways that are affirming and useful. Patients and families receive timely, complete, and accurate information in order to effectively participate in care and decision-making.
*Participation*	Patients and families are encouraged and supported in participating in care and decision-making at the level they choose.
*Collaboration*	Patients, families, health care practitioners, and hospital leaders collaborate in policy and program development, implementation, and evaluation; in health care facility design; and in professional education, as well as in the delivery of care.

*IPFCC is a nonprofit organization whose mission is to “advance the understanding and practice of patient and family-centered care…” IPFCC states that it “accomplishes its mission through education, consultation, and technical assistance; materials development and information dissemination; research; and strategic partnerships.” More information can be found at: www.ipfcc.org

## Results

The number of participants per group and key descriptive information are summarized in Table 2. Focus group participants represented a diversity of ages, races, and ethnicities.

**Table 2:** Focus Group Participant Characteristics

**Patient Groups**				
Characteristic	Region 1	Region 2	Region 3	All Regions Combined
Number of Participants	10	12	13	35
Age (years):				
Range	40–73	20–72	21–69	20–73
Mean	54.2	46.4	47.4	49.0
Sex	7/10 (70%) female	5/12 (42%) female	8/13 (62%) female	20/35 (57%) female
Insurance type				
None/Out-of-Pocket			4 (31%)	4 (11%)
Private	7 (70%)	6 (50%)	6 (46%)	19 (54%)
Private and Medicare	1 (10%)	2 (17%)		3 (9%)
Medicare	2 (20%)		1 (8%)	3 (9%)
Medicaid		2 (17%)	1 (8%)	3 (9%)
Tricare^a^		2 (17%)	1 (8%)	3 (9%)
Race and Ethnicity				
Caucasian	6 (60%)	8 (67%)	9 (69%)	23 (66%)
African American	2 (20%)	3 (25%)	2 (15%)	7 (20%)
Asian		1 (8%)	1 (8%)	2 (6%)
American Indian			1 (8%)	1 (3%)
Hispanic^b^	2 (20%)			2 (6%)
Level of education				
High school		1 (8%)	1 (8%)	2 (6%)
Trade school		1 (8%)		1 (3%)
Some college	1 (10%)	4 (30%)	4 (31%)	9 (26%)
Undergraduate degree	5 (50%)	3 (25%)	6 (46%)	14 (40%)
Professional degree	4 (20%)	3 (25%)	2 (15%)	9 (26%)
**Family Groups**				
Characteristic	Region 1	Region 2	Region 3	All Regions Combined
Number of Participants	12	12	12	36
Age (years):				
Range	31–70	27–69	39–65	27–70
Mean	50.1	50.3	51.2	50.5
Sex	5/12 (42%) female	6/12 (50%) female	5/12 (42%) female	16/36 (44%) female
Race and Ethnicity				
Caucasian	4 (33%)	7 (58%)	6 (50%)	17 (47%)
African American	4 (33%)	2 (17%)	4 (33%)	10 (28%)
Asian	2 (17%)	1 (8%)	1 (8%)	4 (11%)
Hispanic*	2 (17%)	2 (17%)	1 (8%)	5 (14%)
Level of education				
High school			1 (8%)	1 (3%)
Trade school			1 (8%)	1 (3%)
Some college	3 (25%)	5 (42%)	5 (42%)	13 (36%)
Undergraduate degree	3 (25%)	4 (33%)	2 (17%)	9 (25%)
Professional degree	6 (50%)	3 (25%)	3 (25%)	12 (33%)
**Physician Groups**				
Characteristic	Region 1	Region 2^c^	Region 3	All Regions Combined
Number of Participants	8	8	5	21
Age (years):				
Range	38–56	37–69	45–69	37–69
Mean	47.6	36.8	56.6	50.8
Sex	4/8 (50%) female	6/8 (75%) female	2/5 (40%) female	12/21 (57%) female
Race and Ethnicity				
Caucasian	5 (63%)	2 (25%)	3 (60%)	10 (48%)
African American			2 (40%)	2 (10%)
Asian	1 (13%)	5 (63%)		6 (29%)
Unknown	2 (25%)	1 (13%)		3 (14%)
Hispanic	1/8 (13%)	0/7 (0%, 1 unknown)	0/5 (0%)	1/20 (5%, 1 unknown)
Years in practice:				
Range	9–27	8–39	20–27	8–39
Mean	15.9	17.2	23.4	18.3
Number of providers in participant’s practice (including participant)				
1	2/8 (25%)	1/5 (20%)	0/5 (0%)	3/18 (17%)
2–5	1/8 (13%)	1/5 (20%)	4/5 (80%)	6/18 (33%)
6–9	4/8 (50%)	2/5 (40%)	0/5 (0%)	6/18 (33%)
10 or more	1/8 (13%)	1/5 (20%)	1/5 (20%)	3/18 (17%)
Percent of time per week spent in direct patient care in an *outpatient* setting				
Range	40–95	20–100	50–95	20–100
Mean	66.1	72.2	71.1	69.2
Percent of time per week spent in direct patient care in a *hospital* setting				
Range	0–60	0–0	0–31	0–60
Mean	18.4	0.0	5.2	9.6
Percent of time per week spent doing administrative work				
Range	0–27	0–80	5–50	0–80
Mean	15.5	27.8	23.7	21.2

^a^Tricare is the health care program for uniformed services members and their families

^b^Hispanic ethnicity was collected as a non-mutually exclusive category under race

^c^For the Seattle group, only 5 of 8 participants completed forms. Data are for all 8 when the item could be imputed (e.g. sex), or for 5 when imputation was not possible. Data for age are reported for only 4 Seattle participants because of an incomplete form

A key aim of our focus groups was to identify specific and concrete actions that respondents associated with PFCC that could be observed and measured (Table 3). Not all actions fit into the four PFCC concepts in the IPFCC definition (Dignity and Respect, Information Sharing, Participation, Collaboration) so we created an “Other PFCC actions” concept. Actions within the Dignity and Respect concept were the most numerous and most often mentioned in focus groups and will be the primary focus of these results. For the Information Sharing concept, participants identified several information types that physicians and patients share with each other such as test results, explanations of disease processes and treatment procedures, advice regarding health-related issues (e.g. physical activity), treatment options and decision-making advice, and patient education materials as well as the desire for physicians to maximize technology to facilitate communication through the use of texts, secure messaging, and online platforms. The Participation concept included actions that required physicians and patients to interact and share responsibility for outcomes, including shared decision making and health coaching. For Collaboration, participants had limited direct, personal experience with activities that fit in this concept such as patient and family advisory councils and patient advisor participation in quality improvement efforts; the exceptions were filling out patient experience surveys and participating in focus groups. However, participants were interested in the Collaboration concept and had many recommendations for improving collaboration. Two domains, “Advocates for patient needs” and “Coordinates patient care,” and their associated actions were put in the “Other PFCC Actions” concept.

**Table 3:** Summary of Patient- and Family-Centered Care Domains and Actions

Domain	Specific Actions/themes
**PFCC Concept #1: Dignity and Respect**	
Builds relationship	Spends sufficient time
	Listens/gives undivided attention
	Expresses caring and empathy
	Makes personal connection—verbal
	Makes personal connection—nonverbal
	Communicates with honesty and transparency
	Responds without judgement
	Protects privacy
	Relays sense of hope
Personalizes care	Knows the patient
	Includes family
	Understands and accommodates personal circumstances
	Elicits and respects patients’ values
	Practices cultural competence and sensitivity
	Provides empathetic advice
Respects patient and family members’ time	Minimizes wait times
	Notifies patients about delays
	Balances needed time to build relationships with delays in seeing patients
**PFCC Concept #2: Information Sharing**	
Provides patients and family members with information	Provides information—test results and medical records
	Provides information—disease processes and procedures
	Provides information—advice/information regarding a health issue
	Provides information—for decision making/options
	Provides information—patient educational materials and programs
	Helps interpret information
	Follows up
Maximizes technology to communicate	
**PFCC Concept #3: Participation**	
Engages in shared decision making	Elicits patient’s values
	Explores options
	Supports patient and family knowledge, priorities and/or decisions regarding health-related issues
	Refers to alternative healing modalities
Partners with patient	Provides coaching support and advise
	Acknowledges patient’s role and responsibility
	Sets priorities
**PFCC Concept #4: Collaboration**	
Actively seeks out and supports opportunities to collaborate with patient and family members	Solicits input
	Demonstrates commitment to change
	Involves patients and family members in quality improvement
	Provides training and incentives
	Addresses concerns about providing input
Other PFCC Actions	
Advocates for patient’s needs	Advocates with insurance
	Advocates with other clinicians
	Helps find resources
	Helps overcome obstacles to getting medication and/or care
	Engages in persistent problem solving
	Provides uncompensated services
Coordinates patient’s care	Coordinates with other medical staff
	Facilitates communication
	Functions as quarterback for health care team
	Provides quality referrals
Ensures access to care	
Starts with conservative treatment	
Has up-to-date technical knowledge	
Supports wellness and quality of life	

We asked participants to discuss PFCC in relationship to their “doctor’s office or health care center,” but they mainly talked about the actions of their physician or primary care provider. Therefore, we refer to physicians as carrying out PFCC actions although other care team members are also key to implementing PFCC. Related to the importance of all personnel implementing PFCC, we asked participants about openness to team-based care. Most were supportive if all team members exhibited professionalism comparable to their physician.

### Deep dive: Dignity and Respect domains and action themes

We chose to focus on domains and actions in the Dignity and Respect for two reasons: 1) these actions were most frequently raised by participants, and 2) this concept is difficult to translate into observable actions and measures.

#### Builds relationships

The first domain of identified actions was associated almost exclusively with building ongoing patient-physician relationships.

##### Spends sufficient time

This was seen as a key aspect of relationship building and often discussed in relation to both patiently listening and exchanging information.

*The thing I like about [my doctor] the most…she's very thorough and she's patient. And with the VA* [Veterans Affairs]*, she's allocated 18 minutes for each patient; she throws that to the wind, she doesn't care. And she's reprimanded for it a lot of times, she told me, but she'll sit there and listen to you, let's say, up to a half hour. How is everything going in your life…(Patient, Region 3)*

*…when I take a long time with my patients…that’s when everybody is very happy and things are smooth and I felt satisfied with what I did, what the patient did, their family did…Sometimes when you rush through things or you pay attention more to the EHR* [electronic health record] *or want to finish your notes on time, I have done that, too, and when I do that, I don’t feel good about it…(Physician, Region 1).*

##### Listens/gives undivided attention

Being attentive and actively listening to patients and family members was another important action that demonstrated Dignity and Respect. Active listening was perceived as showing that physicians truly understood a patient’s needs. It also validated their perspectives and knowledge.

*He* [my doctor] *listened to you, never tried to tell you what was wrong with you, worked with you to try to establish what was wrong with you, what needed to be done to reach a common level of patient/doctor relationship in the sense that you walked away at least knowing, okay, we both agree that if I have a pain in my toe, it’s in my toe, it’s not in my head. (Patient, Region 3).*

##### Expresses caring and empathy

Another key action theme in Builds Relationship was being outwardly caring and/or expressing caring and empathy to patients. Patient and family members recounted numerous stories of physicians behaving in this way. They felt this was a critical aspect of healing and expressed that it sometimes meant going above and beyond basic job expectations.

*You know, doctors are supposed to care. I mean, that’s what we grew up with…I mean, some of them just act like they don’t care. So I think the best bedside manner is when you make that person who’s obviously in distress or in a crisis in their life, you let them know, “I really care”…I don’t know if that can be trained into somebody, but that’s something that a person needs to have as a human being to another human being, some kind of compassion…(Family, Region 3).*

A physician described a very complex older patient she works with, emphasizing the role of caring in the therapeutic relationship.

*And we see her like every third day or something. Just sit down, kind of hand holding. And she will just come give me a hug. And she was like, “I just feel so much better just seeing you and just feel like somebody’s there who cares for me.” (Physician, Region 2).*

##### Makes personal connection—verbal

Related to, but different from “listening” was connecting with patients more actively through conversation. This included talking respectfully with patients and family members, asking patients about their personal lives, and sharing details about their own, such as hobbies and personal health care experiences.

Well, my primary, when I go to him…he'll stop and pull out a stool, sit down. Ask me how I'm doing, what's going on. And then what I thought was great was he went into his personal life: Well, you know, I'm going to see my parents, me and my wife, and we're going to spend some time there. And I was like, Okay, cool. That's great. So it's not all on me and what's going on with me. He takes the time to tell me what he's doing, you know, with his personal life. (Patient, Region 3)

##### Makes personal connection—nonverbal

Another aspect of making personal connections is using body language and nonverbal cues to establish a caring relationship. Respondents mentioned using eye contact, facial expressions, and appropriate physical touch, such as hand shakes to establish and deepen a respectful and caring relationship. For example, the patient quoted above mentioned the physician who “pulls out a stool and sits down” to physically cue presence and an interest in connecting. The physical examination process was also viewed as another way of non-verbally connecting.

*One of the things we make sure we do is touch everybody.*

*FACILITATOR: Literally put your hands on?*

*PARTICIPANT 3: Human touch. You’d be surprised, the people that see doctors and never get a physical. (Physician, Region 3).*

##### Communicates with honesty and transparency

Respondents identified communicating honestly and transparently as a critical relationship-building action. Trust often builds over time but is related to physicians being open about decision making, to avoid appearing motivated by external forces such as drug companies or personal gain.

*And we know how corporate interest directs pharmaceutical recommendations…So an honest physician can say, “You don’t really need to take these meds. Here’s something that you can do that would really take care of the problem.” And without having to worry about* [name of pharmaceutical company] *breathing down his neck or the hospital making those kinds of demands on these doctors, implicitly or explicitly. (Family, Region 2).*

##### Other relationship-building actions

Several other actions were mentioned less frequently and included “responds without judgment,” “protects privacy,” and “relays a sense of hope.”

#### Personalizes care

The second domain of actions revolve around ways that physicians provide care that is personalized to individual history, needs and preferences.

##### Knows the patient

Focus group participants often stated the importance of really knowing a patient’s health history, family dynamics, personal story and preferences. For example, a family member who responded described great PFCC as, “Knows me by name…You know, knows who I am when I walk in.” (Family, Region 3). Other important aspects of knowing a patient included reading the medical record prior to interacting with the patient, recalling past encounters and taking interest in patients’ goals, values, and life circumstances.

*They should take the time and reread your file so when you come in they don’t say, remind me, what medications are you on again? Or what was the problem the last time? They know what the problem is, especially if it’s chronic and say, okay, yes, I remember you came in and this was a problem, these were the drugs you were on, tell me about your experience. If it’s not working, let’s find a new way to do it. (Family, Region 1).*

Another way physicians got to know a patient was by treating other family members.

*We had a family physician who saw my dad, my mom, all my siblings until he retired…he had an extensive knowledge of our family history on both sides of my mother and father and all the risks involved. And now every time I go to a new doctor…or a new hospital, I have to fill all this stuff out and think back to myself, okay, what do we have…So it would be nice to have…your history on MyChart, the family history. (Family, Region 2).*

##### Includes family

Participants provided numerous examples of how including family was an important element of PFCC and improved care.

*…the PCP* [primary care provider] *that I have now…has requested that next time you come, bring your wife along with you so I can meet your wife and be comfortable and talk with her if it becomes necessary to get to know her for a pertinent matter and also if there’s another family member if I can put on your record who I can talk with. If we find out something, he can get in touch with them. I gave them that information and I really appreciate it…(Patient, Region 1).*

##### Understands and accommodates patients’ personal circumstances

This action involves physicians taking the time to truly understand a patient’s personal circumstances and how they impact their health, treatment choices, and/or ability to follow through with care recommendations. One physician recounted how understanding a patient’s personal circumstances and values was instrumental in allowing the patient to avoid being put in a nursing home.

*Also a patient was placed in a nursing home and everything was done and there were papers to sign. I went there and it doesn’t look like the patient has dementia at all but you can’t challenge the whole family and the doctor who has seen him for several months. But I walked to the patient’s bedside and held his hand and said tell me what you like to do. His eyes brighten and he says “I like to cook”…He started to talk and it was normal. People said he didn’t know what day it was but there was no calendar in his house. If you talk to him, he’s completely normal. The only problem is he was not practicing good hygiene…He did not go to the nursing home, he stayed home just because of a little bit of communication. (Physician, Region 1).*

Respondents also mentioned that home visits were another way physicians and care team members could understand a patients’ social context. One physician told the story of an older woman with multiple chronic conditions who was “going downhill”, emphasizing the benefits of visiting the patient in her home.

*I think something that when you have the time and take the time to understand what’s going on with people, why they’re not getting better when you expect they should be, with everything you’re doing, you really often find out there’s more social kind of things than real medical things. (Physician, Region 3).*

##### Elicits and respects patients’ values

In most groups, participants talked about physicians needing to know and respect patient values, both about their health care preferences and how they wanted to be treated. This ranged from overt willingness to research alternative healing approaches to addressing sensitive issues with consideration for personal preferences.

*My father-in-law had leukemia for about 10 years. And towards the end, he was at* [hospital name]*…he enjoyed taking a shower; but he didn’t want to fall in the shower, and he didn’t want the nurse to be in there with him. So they allowed me– the nurse would stay outside– but they allowed me to go in and help him take a shower. (Family, Region 2).*

##### Practices cultural competence and sensitivity

The need for physicians to be culturally competent was raised by participants in several groups.

*I think it really goes back to what* [Participant 6] *was saying, for me—having multicultural competency and the age thing…I feel like every other time I go to the doctor, it’s…Are you sure you don’t have diabetes? Let’s give you a test for diabetes." It’s like just because I’m a large woman of color does not mean I have diabetes. You know, it’s just like also insensitivity as some doctors, without even reading your…chart…especially with size, I think it’s another like part of the cultural competence is size. Like my mom—one time I took her because she fell. She legitimately fell on the sidewalk and fractured her knee. And the doctor said, “Well, maybe if you weren’t so fat, you wouldn’t have fell.” (Family, Region 2).*

##### Provides empathetic advice

Participants also mentioned wanting physicians to provide advice that was personalized to their specific circumstances and health needs. They often wanted to be told what physicians would do if they had to make similar choices.

*I asked a couple of questions and he said a couple of times, well, for my mom, I would do this and in this situation, in my family, we would have done this or we would do this and that made me a lot more comfortable feeling like do we really need all these invasive things? If he was willing to do that for his mom, obviously, that would be the right choice for us, too. It made me more comfortable and confident in what he was saying. (Family, Region 1).*

#### Respects patients’ and family members’ time

Respecting patients’ and family members’ time emerged as an important issue for many patient and family member participants.

##### Minimizes wait times

Patients and family members felt that long wait times could be interpreted as a sign of disrespect. They raised this issue repeatedly during focus groups as being related to PFCC.

*I know everyone has had that experience where you go in to the doctor, you wait. Even though your appointment was at 2:00, you wait till 2:30. Or the nurse sees you, then you wait till like 3:00 or so like, eventually, it’s like an hour. So they don’t really take into consideration that your time is valuable sometimes. (Patient, Region 2).*

##### Notifies patients about delays

Several groups suggested that physicians might address long waits by developing systems that communicated delays to patients. Suggestions included: using a form that tracks arrival and departure time, text notifications about delays, and systems such as those in restaurants that alert customers about wait times. Participants felt that ambulatory care practices could be more innovative and creative in this area, but emphasized that simple verbal notification would be an improvement over the current situation in many physician offices.

##### Balances time needed to build relationships with delays in seeing patients

Many patients understood and appreciated that sometimes long wait times resulted from clinicians taking the time for relationship building and providing the personalized care they identified as critical to PFCC. Several respondents noted this conflict and talked about the need to balance these two opposing aspects of PFCC—taking the time to build relationships and personalize care vs. respecting patients’ time.

*The time issue is a double-edged sword. As a doctor, I’m sure you want to keep a good timing schedule but then you have situations where like in* [Participant 12]*'s case, he was with him for two hours. That wasn’t planned. You have to have a way of saying I really need to be with you because you’re important to me right now but I have a whole line of people, too. (Family, Region 1).*

## Discussion

We identified specific actions that patients, family members, and physicians associate with PFCC that may be used for measurement and quality improvement purposes, with a particular focus on the concept of dignity and respect. Our data surfaced a detailed list of actions that we organized under IPFCC key PFCC concepts. Not all domains and actions fit neatly under the four concepts. Therefore, several domains were designated “Other PFCC actions.” For this article, we chose to provide a detailed description of domains and actions under the Dignity and Respect concept, a critical aspect of PFCC that is often hard to translate into concrete measures, behaviors, and/or instructions for changes to clinical care. Within the concept of Dignity and Respect, we found three domains of actions: 1) building relationships, 2) providing individualized care, and 3) respecting patients’ time. We identified actions in these domains that break down these abstract ideas into specific and measurable units such as taking time, listening, including family, and minimizing wait times.

While PFCC has been the subject of a great deal of attention in the health services and clinical quality improvement literature, definitions and frameworks continue to be relatively abstract [2, 6, 15, 16, 19, 25–27] and measuring PFCC and associating outcomes [8, 11] continues to be a challenge. Existing measures used to assess PFCC focus primarily on patient satisfaction and/or patient-provider communication, which are overlapping but distinct constructs. We are not aware of any concrete measures of PFCC for primary care settings. Our paper fills this gap by elucidating specific, concrete actions that patients, caregivers, and physicians associated with PFCC. Our broad domains align with and expand upon previous work that focused on concepts such as respect [2, 16, 25], individualized care [16, 19, 26], participation [15, 25] and information sharing [2, 25, 26], while our action themes break these concepts down into discrete actions that can be operationalized and measured.

In addition to the work our team has done to translate these findings into a tool to help outpatient care teams improve and measure the patient and family centeredness of the care they provide, our findings have several other possible contributions to measuring and improving health care. Our action list may be helpful to practices and policymakers in planning and implementing redesign and “transformation” initiatives, specifically in prioritizing actions that patients and family members value and creating workflows and incentives that support care teams in providing PFCC. Finally, our findings could inform training for physicians and other health care workers who have direct responsibility for patient diagnosis, treatment, and ongoing preventive care.

Our study has several limitations. Although we gathered diverse opinions by conducting focus groups in three geographic regions and recruiting participants from a variety of ethnicities, ages, and insurance-coverage groups, our sample was self-selected. We might have missed perspectives not represented among our volunteers who lived primarily in urban and suburban areas. Also, many of the domains and actions were rooted in processes specific to the U.S. health care system and may have limited applicability outside the United States. Finally, for this paper, we chose to focus on the actions of physicians and care teams that contributed to PFCC in ambulatory care settings. However, we acknowledge that PFCC is a partnership between patients and care teams and a similar set of patient actions is likely to facilitate the provision of PFCC in other settings.

## Conclusion

Promoting and facilitating PFCC is a key component of improving health care overall. As U.S. health care systems strive to achieve the triple aims [28], it is essential that we find effective and reliable ways to operationalize and measure PFCC and explore connections between PFCC and patient and clinical outcomes. This is especially true for the concept of dignity and respect in PFCC. Although these terms are frequently mentioned in the literature, no study has operationalized these terms in a way that promotes systematic research and evaluation. Our work is a critical step toward identifying and measuring dignity and respect when providing PFCC in ambulatory care settings. The domains and specific actions presented here are intended to support heath care research and innovations that thoughtfully and deliberately put patients and families at the center of care.

## Additional file

Additional file 1: An additional Word file entitled “Overview Manuscript Supplemental File” provides the recruitment screening questions and focus group guides. (DOCX 33 kb)

## Abbreviations

IPFCC: Institute for Patient- and Family-Centered Care
PFCC: Patient- and family-centered care
