# Supplementary material for: Actions and processes that patients, family members, and physicians associate with patient- and family-centered care
Source: BMC Fam Pract. 2019 Feb 25;20:35. doi: 10.1186/s12875-019-0918-7 (PMC6388493; doi:10.1186/s12875-019-0918-7)
Supplement: Supplementary file 1 — An additional Word file entitled “Overview Manuscript Supplemental File” provides the recruitment screening questions and focus group guides. (DOCX 33 kb) [file 12875_2019_918_MOESM1_ESM.docx]

**I. Recruitment Screening Questions**

**Physician Eligibility Questions**

**Have you been out of a residency or fellowship program for at least one year?**

Yes

No Terminate

**Which of the following best describes the location of your patient care?**

No patient care Terminate

All outpatient

Primarily outpatient with some inpatient

All inpatient Terminate

Primarily inpatient with some outpatient

**Do you consider yourself to be a primary care physician?**

Yes

No Terminate

**II. Focus Group Guides**

**Patient Version**

1. First, let’s find out what brings you here. Please share your name, why you chose to participate in this discussion group, and one thing that will help us remember you (a hobby, interest, how you spend your time).
   *- follow up on each of these, build rapport*
2. I want to remind you that you are here today to talk about your experiences as a patient. So all the questions we ask should be answered from your experiences as a patient.

Now I would like to have you describe a time when you felt your primary care provider or the providers team partnered with you to deliver really great care (we are trying to avoid experiences you may have had in the hospital).

Let’s go around and have you each share your example. We really want to have you be specific about what exactly your doctor or your doctor’s team did that you felt made this experience particularly good.

PROBES *<only asked as needed to encourage richer discussion and/or appropriate for the flow of conversation>*

- 1. What were the specific things that people did that made the experience great?
  2. Why were those actions so important?
  3. What messages, if any, did those actions convey to you?
  4. And did the doctor ask for your input as a caregiver?  Did s/he give you the sense that you had valuable information to share?
  5. How did other members of your doctor’s team or other staff contribute to that experience?
  6. What specifically did the doctor or the doctor’s team do to include you developing the plan for you care?
  7. What specifically did the doctor or the team do to include you in the decision made?
  8. What specifically did the doctor or the team do to include your family member in care planning and decision-making?
  9. To what extent did your provider really assess whether their recommendations made sense in terms of your personal life circumstances, goals and values
  10. What, if any, aspects of the office environment or lay out contributed to your positive experience?
  11. Was there anything else that happened during the appointment that you think affected how you felt about your experience?

1. What were some common themes we heard across your examples regarding what makes care really great and/or how you want your doctor or your doctor’s team to partner with you? <write on flip chart>
2. There is a term that is being used now called, patient- and family-centered care. Patient- and family-centered care means that patients and families are treated with **dignity and respect**; they are provided with **useful information in a helpful manner;** they are **encouraged and supported in participating in care and decision-making**; and they have **opportunities to collaborate in making changes and improvement in health care organizations**. <write bold on flipchart/board ahead of time>

   Now we’d like you to think about this definition in light of the list of specific attributes of great care and partnership you generated. How would do you see your list and the key aspects of this definition fitting together? <point out some of the similarities if they don’t chime in>

PROBES

- 1. What is different?
  2. What would you add to either of these lists from the other?
  3. Are there important distinctions that you think need to be highlighted?

1. To what extent do you feel the health center or practice where you currently you get your care is providing patient and family centered care? What do they do that makes you feel this way?

PROBES

- 1. What do they do that you feel specifically addresses the idea of **dignity and respect**?
  2. What do they do that you feel specifically addresses..**providing using information in a helpful manner**?
  3. What do they do that you feel specifically addresses..**encouraging and supporting participation in your care and decision making**?
  4. What do they do that you feel specifically addresses..**the idea of giving you opportunities to help them make changes and improve care**?

1. Now we are going to focus on your thoughts about your role as a patient. What are the different roles and responsibilities that you feel you have as a patient in health center or practice settings?

PROBES

- 1. What responsibilities do you expect to have to do in your role as a patient?
  2. Have you ever been asked to do things that you didn’t think were your responsibility as a patient? If yes, can you describe them?
  3. What role do you want to play in making care decisions?
  4. How much input should you have in making treatment decisions?
  5. To what extent do you think you as a patient should be expected to follow the directions provided by your doctor and/or the doctor’s team?
  6. What circumstances might lead you to not follow your the instructions provided by your doctor or doctor’s team?
  7. What should you as a patient be expected to do if you feel you aren’t getting good care?
  8. What actions might you as a patient be expected to take to ensure you are getting great health care?

1. How would you feel about getting your care from a team of people that included other clinicians, nurses and other staff, rather than only your doctor?

PROBES

- 1. Has anyone experienced getting primary care from a team? What did you think?
  2. What concerns would you have?
  3. What do you think the benefits would be?

1. Thank you for sharing ways that you have experienced good care at your doctor’s office. We are also interested in hearing your thoughts about improving care so it works better for you.

   What are some of the different ways that you might be interested in working with your health center or doctor’s office, or partnering with them, to improve the care they provide?

   I want to hear what you think about these things:

PROBES

- 1. …fill out surveys?
  2. …participate in a one-time meeting to discuss ideas for improvement?
  3. …participate in a regular meeting (e.g/. that meets monthly) at which the team at your doctor’s office discuss way to improve the care they provide?
  4. What might keep you from participating if your the health center asked you to participate in a meeting or on a committee?
  5. What would be important for a health center or practice to do that would engage your interest, time and energy in helping them improve services?
  6. How much time would you be willing to volunteer in this way without pay?
  7. What things would make it easier for you to participate – e.g., child or respite care, transportation costs, scheduling meetings at convenient times?

1. How would you want your **family members** involved in helping your clinic improve the care they provide?
   PROBES
   1. What unique perspectives do family members provide?
   2. What might be missed if they aren’t included?
2. Some clinics now have patients participate in a monthly meeting about how to improve the quality of care at your health center or doctor’s office, would you be interested in participating in something like this? Why or why not?

PROBES

- 1. What concerns would you have?
  2. What type of preparation or training do you think you might need?
  3. What unique insights would you contribute?

1. As we think about making care more patient-centered, what is one thing you would change? Why?

**Family Member Version**

1. First, let’s find out what brings you here. Please share your name, why you chose to participate in this discussion group, and one thing that will help us remember you (a hobby, interest, how you spend your time).
2. I want to remind you that you are here today to talk about your experiences as a family member or friend helping your loved one with their health care. So all the questions we ask should be answered from that perspective.

Now I would like to have you describe a time when you felt your family member’s doctor or the doctor’s team partnered with you to deliver really great care (we are trying to avoid experiences you may have had in the hospital).

Let’s go around and have you each share your example. We really want to have you be specific about what exactly your doctor or your doctor’s team did that you felt made this experience particularly good.

PROBES *<only asked as needed to encourage richer discussion and/or appropriate for the flow of conversation>*

- 1. What were the specific things that people did that made the experience great?
  2. Why were those actions so important?
  3. What messages, if any, did those actions convey to you?
  4. How did other members of your doctor’s team or other staff contribute to that experience?
  5. What specifically did the doctor or the team do to include you developing the plan for you care?
  6. What specifically did the doctor or the team do to include you in the decision made?
  7. What specifically did the doctor or the team do to include your family member in care planning and decision-making?
  8. To what extent did your provider really assess whether their recommendations made sense in terms of your personal life circumstances, goals and values
  9. What, if any, aspects of the office environment or lay out contributed to your positive experience?
  10. Was there anything else that happened during the appointment that you think affected how you felt about your experience?

1. What were some common themes we heard across your examples regarding what makes care really great and/or how you want your doctor or your doctor’s team to partner with you? <write on flip chart>
2. Now we’d like to shift gears slightly and introduce a definition of patient and family centered care that one of our partner organizations developed based on focus groups similar to this one. I’m going to quickly read this definition.

Patient- and family-centered care means that patients and families are treated with **dignity and respect**; they are provided with **useful information in a helpful manner;** they are **encouraged and supported in participating in care and decision-making**; and they have **opportunities to collaborate in making changes and improvement in health care organizations**. <write bold on flipchart/board ahead of time>

We would like to you talk about what your family member’s doctor or doctor’s office does that demonstrates these concepts to you. There may be some overlap with our list or you might think of other things you would add. Let’s go through each of these concepts…What does your family member’s doctor/doctor’s office do that demonstrates…

PROBES

- 1. …dignity and respect
  2. …providing using information in a helpful manner?
  3. …encouraging and supporting participation in your care and decision making
  4. …the idea of giving you opportunities to help them make changes and improve care
  5. What do they do that makes you feel this way?
  6. What else do they do that makes you feel like a partner?
  7. Do they solicit feedback from you about how to make the care they provide better? If so, how?
  8. Do you know if there is a patient or family advisory council or group at the health center or practice or other ways they ask patients and families to become involved in helping improve the health center or doctor’s office?
  9. What do they do that gets in the way of you feeling like a partner?
  10. To what extent does your health care provider tailor recommendations they make to your particular life circumstances (such as transportation limitations, etc)?

1. What are some other ways that you might expect/want your family member’s doctor/ doctor’s office to show that they are providing PFCC?

PROBES

- 1. What could they do that would promote your feeling they were treating you and your family member with **dignity and respect**?
  2. What could they do that would promote your feeling they were **providing useful information in a helpful manner?**
  3. What could they do that would promote your feeling they were encouraging and supporting **participation in your care and decision making**
  4. What could they do that would promote your feeling they were the idea of giving you and your family member **opportunities to help them make changes and improve care**
  5. What could they do during your **in person visits** that makes you feel like you are a partner in your care?
  6. **Outside of the in person visits** with the doctor what could the practice do that promotes a sense of partnership and collaboration?
  7. What types of information do you want from the practice? What types of reminders are useful?
  8. How should this **information be shared** (e.g., email, written documents, phone, etc)?
  9. What **resources** would you find useful, e.g., education classes, peer support meetings, video or written materials?

1. Now we are going to focus on your thoughts about your role as a patient’s family member. What are the different roles and responsibilities that you feel you have as a patient in health center or practice settings?

PROBES

- 1. What do you expect to have to do in your role as a family member?
  2. Have you ever been asked to do things that you didn’t think were your responsibility as a family member? If yes, can you describe them?
  3. What role do you want to play in making care decisions?
  4. How much input should you have in making treatment decisions?
  5. To what extent do you think you as a family member should be expected to follow the directions provided by your doctor and/or the doctor’s team?
  6. What circumstances might lead you to not follow your the instructions provided by your family member’s doctor or doctor’s team?
  7. What should you as a family member be expected to do if you feel you aren’t getting good care?
  8. What actions might you as a family member be expected to take to ensure you are getting great health care?

1. How would you feel about your family member getting care from a team of people that included other clinicians, nurses and other staff, rather than only your doctor?

PROBES

- 1. Has anyone experienced getting primary care from a team? What did you think?
  2. What concerns would you have?
  3. What do you think the benefits would be?

1. We are interested in specific way that family members can partner with their doctors and doctor’s offices to improve care. What are some of the different ways that you might be interested in partnering and giving feedback?

PROBES

- 1. …fill out surveys?
  2. …participate in a one-time meeting to discuss ideas for improvement?
  3. …participate in a regular meeting (e.g/. that meets monthly) at which the team at your family member’s doctor’s office discuss way to improve the care they provide?
  4. What type of preparation or training do you think you might need?
  5. What unique insights would you contribute?
  6. What might keep you from participating if your the health center asked you to participate in a meeting or on a committee?
  7. What other ideas do you have for giving feedback?
  8. What could they do to encourage you to participate in these types of activities?
  9. What things would make it easier for you to participate – e.g., child or respite care, transportation costs, scheduling meetings at convenient times?
  10. What types of activities would you be most interested in participating in?
  11. How much time would you be willing to volunteer in this way without pay?

1. As we think about making care more patient-centered, what is one thing you would change? Why?
2. What other thoughts do you have around the issue of providing more patient- and family -centered care and/or getting individuals more involved in their health care and the improvement of health care?

**Provider Version**

1. First, we would like you to introduce yourselves. Would you briefly tell us: 1) your first name or what you would like to be called, 2) what type of medicine you practice (family medicine, internal, OB, etc.), 3) why you chose to participate in this discussion group, 3) and one thing that will help us remember you (a hobby, interest, how you spend you time).
2. Now I would like to have you think about a time when you felt you and/or your team partnered with a patient and/or their family in a way that resulted in really great care. Take a few minutes to either jot down some notes about that experience. We are looking for an experience that goes beyond making a difficult diagnosis, but one that might have been difficult but that you handled particularly well.

Underline the specific actions you took to better partner with the patient and their family.

Let’s go around and have you each share your example. We really want to have you highlight the things that happened you felt made this experiences particularly good.

PROBES

- 1. What were the specific things that you or others did that made the experience great?
  2. Why were those actions so important?
  3. What, if any, other messages did those actions convey?
  4. How did other people in the health care center or doctor’s office contribute to that experience?
  5. What happened during the appointment that you think affected how you felt?
  6. What specific things did you do that helped you partner with your patient?
  7. What, if any, aspects of the office environment or lay out contributed to to making this a positive experience for your patient?
  8. Was there anything else that happened during the appointment that you think affected how you felt about this experience?
  9. Was there anything else that happened during the appointment that you think affected how your patient felt about this experience?

1. Now that you have had a chance to think about experiences that were great, I’d like to have us brainstorm some of the common themes that emerged around what it looks like when you really partner with patients to provide them great care. What are some of things that—from your perspective—**you and your team do** to make the care provided really great? <write up on flip chart/board>
2. Now we’d like to shift gears slightly and introduce a definition of patient and family centered care that one of our partner organizations developed based on focus groups similar to this one. I’m going to quickly read this definition.

   Patient- and family-centered care means that patients and families are treated with **dignity and respect**; they are provided with **useful information in a helpful manner;** they are **encouraged and supported in participating in care and decision-making**; and they have **opportunities to collaborate in making changes and improvement in health care organizations**. <write bold on flipchart/board ahead of time>

   We would like to you talk about what you do that demonstrate these concepts to your patients and their families. There may be some overlap with our list or you might think of other things you would add. Let’s go through each of these concepts…What are some very specific actions you and/or your team carry out that demonstrate…

   PROBES
   1. …**dignity and respect**
   2. **…providing using information in a helpful manner?**
   3. **…encouraging and supporting participation in your care and decision making**
   4. **…the idea of giving you opportunities to help them make changes and improve care**
   5. What do you do that makes you believe make your patients and their families feel this way?
   6. **Do you ask for your patient’s input during your encounters with them? If so, how to do solicit their input? Do you have some standard ways you ask them for input?**
   7. **Do you solicit feedback from patients and family members about how to make the care you provide better? If so, how?**
   8. Do you know if there is a patient or family advisory council or group at the health center or practice or other ways they ask patients and families to become involved in helping improve the health center or doctor’s office?
   9. What do they do that gets in the way of you feeling like a partner?
   10. To what extent does your health care provider tailor recommendations they make to your particular life circumstances (such as transportation limitations, etc)?
3. What are some of the different ways that your health care center or office has patients and family members involved in improving the care your health care center or office provides? For example, do you have a patient advisory group?

PROBES

1. For example, do you have a patient advisory group?
2. …do patients fill out patient experience surveys?
3. …do they participate in a standing quality improvement meeting?
4. …help with interviewing and/or hiring new staff?
5. …help create a new staff position or new workflow?
6. …help create or give input on new educational materials?
7. …give input on a clinic remodel?
8. What lessons have you learned about getting patients and family members involved?
9. How do you think partnering with patients and family members in these ways has changed and/or improved the care your /practice/office provides?
10. How much time have you found that patients and family members are willing to volunteer in these activities?
11. How much, if anything, does the practice or office pay patients and/or family members for participation?
12. What, if anything, do you think you would need to do to prepare patients and or family members to productively partner?
13. What, if anything, do you think you would need to do to prepare clinicians and staff to effectively partner?
14. Are there activities that you feel patients and family members really would not or should not do? What would those be?
15. What concerns do you have?
16. How might you measure how well you are doing partnering with patients and family members? Can you think of a possible metric/measure that would work in your office/practice?
17. If patients and/or family members participated in a monthly quality improvement meeting as a family member, what do you think they could contribute to your QI work?
18. What unique insights would they bring?
19. What are some other ways – some specific actions - that you **might consider making to increase the extent to which your care** PFCC? [IDEAS FOR IDEAL FUTURE]

PROBES

1. What are some other things you could do that would promote your patients feeling that they are being treated with **dignity and respect**?
2. What are some other things you could do that would promote your patients feeling that they were **provided useful information in a helpful manner?**
3. What are some other things you could do that would promote your patients feeling that they encouraged and supported to **participate in their care and decision making**
4. What are some other things you could do that would promote your patients feeling that they were given **opportunities to make changes and improve care**
5. What are some other things you could do **in person visits** that would make patients and family members feel more like partners in their care?
6. **Outside of the in person visits** what are some other things you could do to promote a sense of partnership and collaboration?
7. How should this **information be shared** (e.g., email, written documents, phone, etc)?
8. What **resources** could you provide that patients might find useful, e.g., education classes, peer support meetings, video or written materials?
9. What, if anything, do you think you would need to do to prepare patients and or family members to productively partner?
10. What, if anything, do you think you would need to do to prepare clinicians and staff to effectively partner?
11. Are there activities that you feel patients and family members really would not or should not do? What would those be?
12. What concerns do you have?
13. How might you measure how well you are doing partnering with patients and family members? Can you think of a possible metric/measure that would work in your office/practice?
14. If patients and/or family members participated in a monthly quality improvement meeting as a family member, what do you think they could contribute to your QI work?
15. What unique insights would they bring?
16. To what extent have you implemented a team-based model of care in your practice?
17. How does your team help you provide great care and partner with patients?
18. To what extent do you feel the health center or practice where you currently work provides patient and family centered care? What do they do that makes you feel this way?
19. Now we are going to focus on your thoughts about the role that patients and family members can and do play in their health care. What are the different roles and responsibilities that you feel patients and family members have in outpatient settings?

PROBES

- 1. What do you expect patient and family members to do when it comes to partnering with you around their care?
  2. Do you ever ask patients or family members to take on responsibilities that they don’t appear to want to take on?
  3. What do you expect the patient to do when it comes to health care?
  4. To what extent do you think patients should be expected to always follow the directions you provide?
  5. What circumstances might lead your patients to not follow your instructions?
  6. What should patients and/or family members be expected to do if they feel they aren’t getting good care?
  7. What actions might you expect patients and/or family members to take to ensure they are getting quality health care?
  8. How do you partner with patients and family members when they bring in information that they have researched themselves? Do you have any concerns about patients doing this?

1. As we think about making care more patient and family-centered, what is one thing you would change? Why?
2. What other thoughts do you have around the issue of providing more patient- and family centered care?
